# Smelters and Mortality

**DOI:** 10.1289/ehp.10447

**Published:** 2007-09

**Authors:** George M. Hidy

**Affiliations:** Envair/Aerochem Placitas, New Mexico, E-mail: dhidy113@comcast.ne

Pope et al. (2007) provide results for reduced mortality during the 1967–1968 smelter strike in the U.S. Southwest. They ascribed mortality reduction to a decrease in ambient sulfate from the smelters. Although I found the thesis interesting, there is confounding that should be noted involving *a*) inconsistencies in state mortality relationships; *b*) the trace metal role, and possibly carbon exposure from the plant complexes; and *c*) ambiguities associated with SO_4_ sampling.

The basis for the study by Pope et al. (2007) is a study by Trijonis and Yuan (1978), who analyzed the National Air Surveillance Network (NASN) SO_4_ and visibility. They attributed improved visibility across the Southwest to SO_4_ reduction during the strike. Ambient SO_4_ includes SO_4_ from oxidation of sulfur dioxide in air (secondary) and that emitted directly (primary). NASN data [e.g. U.S. Environmental Protection Agency (EPA) 1971, 1972, 1973] indicate a strike reduction in SO_4_ (0.1–3.6 μg/m^3^) at sites in the region (Trijonis and Yuan 1978; Table 16) and not Pope et al.’s uniform 2.5 μg/m^3^. However, the accompanying association with non–weather-related visibility change is problematic (e.g., Hidy 1984).

If the smelter SO_4_ was present regionally, exposure to concentration gradients of SO_4_, SO_2_, and metals would be expected with distance from the plants (Eldred et al. 1983; Malm et al. 1990). Pope et al. (2007) did not differentiate their results by distance from the smelters, but some information in their article is relevant because mortality is associated mainly with population centers (cities).

The risk estimates for New Mexico presented by Pope et al. (2007) in their Figure 6 (dominated by Albuquerque; the nearest smelter is 300 km south southwest) show high levels of mortality reduction in spite of an increase in annual average SO_4_ between 1966 and 1967–1968 and a negligible reduction during the strike. The mortality reduction in Nevada is largest of the four states presented by Pope et al. 2007 (Figure 6), even though Reno and Las Vegas (population centers) are upwind of the smelters and are far distant over mountain ranges from the nearest smelter at Ely. The smallest risk change is in Arizona, but reductions in Utah are similar to those of New Mexico. However, note that the population centers in Arizona (Phoenix and Tucson) and Utah (Salt Lake City) are close to smelters.

The results of Pope et al. (2007) are further confounded by the fact that trace metals and carbon accompany the emissions from plant complexes (e.g., Leipold and Chow 1998; Small et al. 1981). Local exposure to smelter emissions involves primary SO_4_ as well as SO_2_ [the apparent SO_4_ concentrations are biased high by a variable SO_2_ filter adsorption artifact (e.g., Lee and Wagman 1966; Lipfert 1994)]. Emission reduction would reduce SO_4_, including the bias from SO_2_ adsorption; metals such as copper, lead, iron, cadmium, antimony, chromium, nickel, and arsenic; and possibly carbon. Distant exposure would be enriched in secondary SO_4_ up to a point, followed by decline from atmospheric dilution and deposition. Pope et al. (2007) mentioned the metal–SO_4_ linkage but did not explore it relative to the SO_4_ theory. The combined exposure in sulfur oxides and metals from the smelters preset in aerosols from many sources adds further complexity to interpreting their results, including the differences in Salt Lake City and the Arizona cities.

Recent research suggests that a combination of primary SO_4_ and metals from oil combustion, as well as carbon emissions from motor vehicles, may be important factors in mortality risk (e.g., Grahame and Schlesinger 2007). The smelter inferences appear inconsistent with these findings.

Pope et al. (2007) focused on the strike in the Southwest. However, they excluded the same period in Montana as a cross-check on their results. In the 1960s, a major copper production complex was located in Anaconda, Montana. NASN data from nearby Helena and Glacier National Park do not show a significant change in average SO_4_ concentrations during the strike years (U.S. EPA 1971, 1972, 1973). This appears inconsistent with the widespread reductions seen in the Southwest. This difference could be a valuable adjunct to their results if mortality data are available.

Pope et al. (2007) provided a “natural” experiment in regional sulfur oxide (and metals) reduction. Their results should be examined further to insure that their interpretation is robust.
